# Preliminary recommendations for lung surgery during COVID‐19 epidemic period

**DOI:** 10.1111/1759-7714.13423

**Published:** 2020-04-14

**Authors:** Xin Li, Minghui Liu, Qingchun Zhao, Renwang Liu, Hongbing Zhang, Ming Dong, Song Xu, Jinghao Liu, Honglin Zhao, Sen Wei, Zuoqing Song, Gang Chen, Jun Chen

**Affiliations:** ^1^ Department of Lung Cancer Surgery Tianjin Medical University General Hospital Tianjin China

Novel coronavirus pneumonia (NCP), which has swept across China and emerged in several countries around the world since December 2019, has attracted global attention. NCP has had a huge impact on the world's medical, political and economic sectors. Since March, under the efforts of the Chinese government, people and the guidance of “New Coronavirus Pneumonia Diagnosis And Treatment Program (Trial Version 7)” issued by The National Health Commission, PRC (http://www.nhc.gov.cn/yzygj/s7653p/202003/46c9294a7dfe4cef80dc7f5912eb1989.shtml), the epidemic situation has been effectively controlled and the number of people cured is now rising. On 12 February 2020, the World Health Organization (WHO) officially named the disease caused by the virus COVID‐19. The pathogen of the new coronavirus is a novel coronavirus, initially known as 2019 new coronavirus (2019‐ncov), which was subsequently uniformly named SARS‐CoV‐2 by The International Committee on Taxonomy of Viruses (ICTV). Currently, it is believed that SARS‐CoV‐2 belongs to the family coronavirus with enveloped, round or oval particles, often pleomorphic, with a diameter of 60–140 nm. SARS‐CoV‐2 has the characteristics of human‐to‐human transmission and strong infectivity. The main route of transmission is respiratory droplets and contact, aerosol and digestive tract. The incubation period is 1–14 days, mostly 3–7 days. Fever, fatigue and dry cough are the main manifestations. A small number of patients have nasal congestion, runny nose, sore throat and diarrhea and other symptoms. In severe cases, dyspnea and/or hypoxemia usually occur one week after the onset of the disease, and in severe cases, it rapidly progresses to acute respiratory distress syndrome, septic shock, metabolic acidosis that is difficult to correct, and bleeding and coagulation dysfunction. It is worth noting that patients with severe and critical illness may have moderate and low fever, or even no significant fever. Laboratory examination shows that the total number of white blood cells in peripheral blood in the early stage of disease is normal or decreased, while the lymphocyte count is decreased. In some patients, liver enzymes, LDH, myoenzyme and myoglobin are increased. Elevated troponin is seen in some critically ill patients. In most patients, C‐reactive protein (CRP) and erythrocyte sedimentation rate are elevated, and procalcitonin is normal. In severe cases, D‐dimer increases and peripheral blood lymphocytes progressively decrease. Nucleic acid amplification tests (NAATs) and other methods are used to detect coronavirus and nasopharyngeal swabs, sputum samples, lower respiratory tract secretions, blood, feces and other specimens are collected for laboratory analysis. Most patients have a good prognosis, while a few are in critical condition. The elderly and those with chronic underlying diseases have a poor prognosis. Cases in children have been reported to be relatively mild.^1^

In order to prevent the further spread of the epidemic, many countries and regions around the world have initiated measures such as income and expenditure restrictions, traffic restrictions and closed community management to reduce the spread of the virus. In this severe situation, the diagnosis and treatment process of patients with lung space‐occupying lesions is inevitably affected. Surgery can be postponed for some benign lesions due to their slow growth, but for patients with malignant pulmonary lesions, delayed treatment may lead to tumor progression, tumor metastasis, prognosis or even life‐threatening adverse consequences. Thoracic surgeons around the world should work together to standardize management, streamline procedures, and develop procedures for diagnosis and treatment during epidemics. Meanwhile, this recommendation should be updated according to the changes as they evolve in the epidemic situation and as further understanding of the disease is acquired. We have previously published a paper on preliminary recommendations for pulmonary surgery in China during the SARS‐CoV‐2 pneumonia epidemic.^2^ Based on the experience of Chinese thoracic surgeons, we hereby present the following preliminary suggestions to thoracic surgeons worldwide for extensive discussion and reference.

• Patients with lung space‐occupying lesions are encouraged to seek treatment nearby and locally, so that patient transport and personnel flow are minimized.

• Patients with lung space‐occupying lesions with respiratory symptoms such as fever, cough, and wheezing should first be diagnosed at a fever clinic designated by the local government. If they are suspected of having SARS‐CoV‐2 infection, they should be admitted to a designated isolation ward. After SARS‐CoV‐2 infection has been excluded and space‐occupying lesions in the lungs confirmed by computed tomography (CT) scan, they could be transferred to thoracic surgery for further diagnosis and treatment.

• PET‐CT or percutaneous pulmonary puncture biopsy indicates benign lesions. If patients need elective surgical treatment, it is recommended for them to be followed up outside the hospital for observation (no less than three months), and a surgical treatment chosen after the outbreak is over or relatively stable.

• PET‐CT or percutaneous pulmonary puncture biopsy indicates malignant lesions. If the tumor is of central type (bronchoscopy is not recommended during the outbreak prevention and control period), patients can be recommended to receive neoadjuvant therapy first, and choose the surgical treatment after the outbreak is over or relatively stable.

• Patients with central malignant lesions accompanied by massive hemoptysis (not confined to hemoptysis in special periods, which should be determined in combination with the general situation of patients), or patients with major airway involvement accompanied by severe dyspnea and in critical condition, and those patients who can be completely removed from the technical perspective after discussion in the department can be treated with emergency surgery.

• For peripheral solid nodules with a diameter of less than 3 cm considered as malignant lesions by PET‐CT or percutaneous pulmonary puncture biopsy, short‐term regular follow‐up (once a month) can be recommended during the outbreak prevention and control period. If the nodules increase by more than 20% during the follow‐up period, surgical treatment should be considered. If the diameter of the nodules is ≥3 cm, surgical treatment should be considered.

• Lung ground‐glass nodules (GGNs). According to the results of the national lung screening test, the malignant risk of chest CT review of 4–5 mm GGNs is 0.4% annually. The risk of malignancy for CT re‐examination every six months is 1.1%, 3.0%, and 5.2%, respectively. The risk of malignancy is 10.9% for chest CT re‐examination every three months with a nodule diameter greater than, or equal to, 20 mm.^3^ Therefore, we suggest that during the epidemic prevention and control period, whether the patient has pure GGNs, mixed GGNs or multiple GGNs (SARS‐CoV‐2 infection should be excluded for multiple GGNs), follow‐up re‐examination should be the main recommendation, and surgery should not be carried out. For those with nodules 5 mm or less in diameter, they should be re‐examined again in less than one year; for those with nodules with a diameter of 6–19 mm, they should be re‐examined six months later; for those with a diameter of ≥20 mm, they should be re‐examined less than than three months later to reduce the risk of cross infection.^4^ At the same time, great attention should be paid to the pulmonary imaging features of new coronary pneumonia, which are ground‐glass changes in the lungs. For newly discovered GGNs or space‐occupying lesions in the lungs, follow‐up should be conducted for at least three months to exclude the possibility of pulmonary changes in new coronary pneumonia.

In general, conventional pulmonary surgery can be divided into two categories: (i) Elective surgery: defined as operations that can be observed over three months, including various GGNs <3 cm in diameter, benign probable nodules or space‐occupying lesions in the lung with a probability of more than 70%, and benign pulmonary diseases that can be treated conservatively, such as bronchiectasis with hemoptysis, etc. Surgery is not recommended or a careful decision should be made during the epidemic period. (ii) Limited period surgery: defined as the operation should be performed within one month, including patients with a clear diagnosis of lung cancer, or patients with a malignant lung tumor mainly considered in the clinical diagnosis of various GGNs >3 cm or above. Such surgery should only be performed on the basis that the possibility of a patient having new coronary pneumonia has been eliminated. Various types of emergency pulmonary surgery are not discussed in this article.

During the unstable epidemic period, if patients need routine thoracic surgery, preoperative screening for SARS‐CoV‐2 infection may include CT imaging in patients with suspected infection or fever, cough, muscle soreness, fatigue, coughing up phlegm, headache, diarrhea, or any one of the symptoms, and should be carried out in accordance with suspected cases. It is also feasible after a SARS‐CoV‐2 test to rule out infection for a patient to be placed in an isolation ward for 1–14 days after surgery. Before the proposed surgery, routine blood tests, CRP, and a meeting to review the chest CT, should be carried out to determine whether the operation is safe to proceed. During the operation, isolation and protection measures should be increased to prevent the occurrence of cross‐infection. For patients undergoing emergency thoracic surgery, if the symptoms mentioned above occur, isolation measures should be taken during the operation, and the possibility of SARS‐CoV‐2 latent infection should be eliminated without delay. At the same time, a SARS‐CoV‐2 test should be conducted. After the test results are available, the postoperative direction of patients can be decided. Patients suspected to be infected should be treated in an isolation ward, while those patients with negative results can be treated in a common ward.

During the perioperative period, monitoring the patient's body temperature twice a day is recommended, as well as taking blood for CRP, routine blood test, liver and kidney function on the first day after the operation and then the patient should be re‐examined once every three days, and the chest CT re‐examined regularly after the operation in one week. In the case of fever (>37.3°C), symptomatic treatment should first be given, and CRP and blood routine test urgently checked at the same time, and conventional treatment should be continued after the new coronavirus infection has been excluded. If the fever situation does not improve after 48 hours, and conventional postoperative fever factors, such as effusion and common pathogen infection at the surgical site are excluded, the possibility of new coronary pneumonia infection should again be ruled out, and appropriate treatment given. The hospital stay of postoperative patients should be shortened to avoid cross infection.

In our proposal, the nucleic acid test of SARS‐CoV‐2 is not taken as the gold standard for positive or negative new coronavirus. The main reasons are as follows: (i) although there are many nucleic acid detection techniques for SARS‐CoV‐2 at present, they have not been verified, there is a lack of unified standards, the specificity and sensitivity are variable, and in particular the sensitivity is unknown; (ii) in clinical practice, there have been a certain proportion of false negative results in early nucleic acid testing in some patients, especially those who have repeatedly tested negative in early nucleic acid testing and were finally diagnosed as positive infection cases. Therefore, epidemiological exposure history, clinical characteristics, routine biochemical test containing CRP and comprehensive review of chest CT are important judgment bases before pulmonary surgery is considered. The isolation and observation period of two weeks before the operation is helpful to further exclude the possibility of new coronary pneumonia. Finally, it is hoped that our suggestions will serve as a guide for more thoracic surgeons to participate in the discussion, so as to form a standard with certain consensus, which will not only protect the interests of patients, but also reduce the infection of medical staff.^5^

## Disclosure

The authors declare that there are no conflicts of interest.

## References

[1] Chen N , Zhou M , Dong X *et al*. Epidemiological and clinical characteristics of 99 cases of 2019 novel coronavirus pneumonia in Wuhan, China: A descriptive study. Lancet 2020; 395: 507–13. 32007143 10.1016/S0140-6736(20)30211-7 PMC7135076

[2] Li X , Liu M , Zhao Q *et al*. Preliminary recommendations for lung surgery during 2019 novel coronavirus disease (COVID‐19) epidemic period. Zhongguo Fei Ai Za Zhi 2020; 23 (3): 133–135. 32077440 10.3779/j.issn.1009-3419.2020.03.01 PMC7118336

[3] Robbins HA , Katki HA , Cheung LC , Landy R , Berg CD . Insights for management of ground‐glass opacities from the National Lung Screening Trial. J Thorac Oncol 2019; 14 (9): 1662–5. 31125735 10.1016/j.jtho.2019.05.012 PMC6909540

[4] Hammer MM , Palazzo LL , Kong CY , Hunsaker AR . Cancer risk in subsolid nodules in the National Lung Screening Trial. Radiology 2019; 293 (2): 441–8. 31526256 10.1148/radiol.2019190905 PMC6823608

[5] Liang W , Guan W , Chen R *et al*. Cancer patients in SARS‐CoV‐2 infection: A nationwide analysis in China. Lancet Oncol 2020; 21 (3): 335–7. 32066541 10.1016/S1470-2045(20)30096-6 PMC7159000

